# Factors Contributing to Maternal-Child Separation in Port-Au-Prince, Haiti

**DOI:** 10.5334/aogh.2640

**Published:** 2019-11-13

**Authors:** Jessica Ashley, Ariana Johnson, Hiwot Woldu, Craig L. Katz

**Affiliations:** 1 Department of Medical Education, Icahn School of Medicine at Mount Sinai, New York, US; 2 University of Miami Department of Public Health Sciences, US; 3 Department of Psychiatry, New York University School of Medicine, US; 4 Departments of Psychiatry and System Design and Global Health, Icahn School of Medicine at Mount Sinai, New York, US

## Abstract

**Background:**

The Haitian orphanage sector receives more than 70 million United States Dollars (USD) in foreign aid annually and continues grow; there are over 500 orphanages in Port-Au-Prince alone. An estimated 80% of the 30,000 children living in Haitian orphanages have at least one living parent.

**Objectives:**

This research seeks to identify factors contributing to maternal-child separation in Port-Au-Prince to understand motivations and attitudes surrounding maternal-child separation. We hypothesized that poverty, health status, and current state of the family unit are influential in the mother’s decision to separate from her child.

**Methods:**

From June to August 2017, a chain referral sampling study was conducted in Port-Au-Prince and the surrounding metropolitan area. Seventy interviews were conducted with (1) Mothers – separated (n = 8) and non-separated (n = 48) – and (2) Community leaders exposed to maternal-child separation (n = 18). The semi-structured interview consisted of questions about (1) exposure to maternal-child separation, (2) circumstances surrounding maternal-child separation, and (3) factors contributing to maternal-child separation. Additionally, all mothers completed a survey including demographic information and multiple validated surveys: Maternal Postpartum Quality of Life, Patient Health Questionnaire (PHQ-9), and PTSD Checklist (PCL) to address quality of life, depression, and PTSD, respectively.

**Findings:**

This study found separation to be associated with poor economic means compounded with other factors, most notably access to education, ability to care for disabled children, insufficient support, and poor maternal mental health. Additional themes identified include negative stigma towards maternal-child separation and sparse education surrounding family planning. Quantitative findings revealed separated mothers experienced significantly higher rates of PTSD compared to non-separated, small but statistically worse quality of life, and no difference in rates of major depression.

**Conclusion:**

This research finds economic means to be insufficient in predicting maternal-child separation, with access to education being the most salient contributing factor mentioned after economics. Findings from this study will inform development of programming focusing on education, family planning, and social support in Port-Au-Prince.

## Background

An estimated 30,000 children live across 750 orphanages in Haiti, with over 500 orphanages in Port-Au-Prince alone. The Haitian government estimates 80% of these children have at least one living parent and nearly all have other family members. Despite this, the orphanage sector continues to thrive [1].

Developed countries that fund the orphanage sector, including Australia, European countries, and the United States, have themselves departed from orphanage-based care in favor of family-based foster systems [1]. In 1989, the United Nations Convention on the Rights of the Child established it is within a child’s rights to “grow up in a family environment [2,3].” This conclusion concurs with research showing lifelong physical and psychological impairments in children raised in orphanage care. For over 80 years, studies have documented developmental delays in children separated from family environments and placed in orphanages or other institutional care. In 2015, researchers found deficits in physical growth, cognitive function, neurodevelopment, and social-psychological health in children raised in institutional care, with deficits more pronounced in those with less access to individualized care [2]. In addition to developmental concerns, of the Haitian orphanages assessed, 140 of the orphanages have poor living conditions that leave children at high risk of violence, exploitation, neglect, or even avoidable death [1]. At best, well-run orphanages attempt to provide a family-like environment, but the secure attachments and support of family-based care are rarely achieved. At worst, children in orphanages suffer due to intolerable living conditions.

The outcomes of orphanage care are well established, but the Haitian orphanage sector continues to grow; contradicting international trends, UN child protection conventions and standards, and the Haitian Government’s national plan to reduce reliance on orphanage care and decrease human trafficking. Economic and political instability, poverty, foreign intervention, and natural disasters have influenced the current environment where it is not uncommon for Haitian women to struggle to provide for their children [1]. Following the earthquake of 2010, many children were displaced and an internationally perceived “orphan crisis” ensued [1,4]. Rather than placing focus on sustainable solutions, the emergent nature of the problem led to a 150% increase in the number of orphanages in Haiti. International support of the Haitian orphanage sector has endured. International – largely North American – non-governmental organizations (NGOs) provide 70 million USD to only one-third of Haitian orphanages. It is estimated that over 100 million USD in total goes into the Haitian orphanage sector annually, making this sector one of the most significant recipients of international aid to Haiti. This number does not account for additional funding from churches, cash, in-kind donations, and other non-reportable forms of support [1].

The size and wealth of the orphanage sector overpowers L’Institut du Bien-Etre Social et de Recherches (IBESR) – the Haitian government agency responsible for child welfare – as the agency’s annual budget is less than 750,000 USD. In 2015, IBESR adopted a 2016–18 strategy to reduce reliance on orphanage care and enforce regulations on orphanages. IBESR has been able to officially register 15% of orphanages and close several unsafe orphanages, but its capacity to monitor orphanages and enforce regulations is limited [5]. Efforts to shift towards family-based care are impaired by the limited resources of IBESR relative to the orphanage sector. The rapid growth of the orphanage sector continues to overwhelm local child protections efforts in Haiti, creating a largely unregulated system that leaves thousands of children at risk [1].

Given the lifelong consequences of orphanage care, the number of children in orphanages with living parents, and the limited capacity of the Haitian government to monitor the precarious living environments of these children, our research focuses upstream to learn about the condition of mothers and what factors influence their decision to separate from their children. This research seeks to elucidate factors contributing to maternal-child separation in Port-Au-Prince to better understand the motivations and attitudes surrounding separation with the hopes of influencing policies and practices geared towards prevention of maternal-child separation. We focus on maternal-child separation as primary caregivers are largely Haitian mothers [1].

We acknowledge the significance of the language utilized to discuss families who experience maternal-child separation. While conducting interviews, we found the word “abandonment” was used to describe instances of maternally-initiated separation from children, though the word itself carried a significant burden of stigma, as will be discussed. The concept of a mother giving up the role of primary caretaker translates to “abandone” in Kreyol, and as such, this language is reflected in transcribed interviews. Similarly, translation of study interview questions into Kreyol utilized “abandone” as study participants repeatedly expressed lack of understanding of direct translations of the words “separation” and “relinquishment” in this context. In order to be respectful of study participants and refrain from introducing additional stigma to their experience, we will use the language “separation.” For the sake of this study, maternal-child separation is defined as having a child live full-time in an orphanage (with or without continued parental contact), having a child live in another city (with or without continued parental contact), or having no contact with one’s child. To our knowledge, no research of this kind has been done in Haiti. We hypothesized that poverty, along with health, and status of the family unit are influential in maternal-child separation.

## Methods

### Design and Sampling

From June to August 2017, a chain referral sampling study was conducted in Port-Au-Prince and the surrounding metropolitan area. Participants were community leaders, non-separated, and separated mothers. The research employed both quantitative and qualitative methods to allow mothers and community leaders to share their understandings of maternal-child separation and to understand the context in which such crucial decisions are made.

We conducted semi-structured interviews surrounding perceived factors contributing to maternal-child separation and maternal stress in order to capture the experiences of mothers (Table 1) and community leaders (Table 2) living in the Port-Au-Prince metropolitan area. Qualitative information was collected in face-to-face semi-structured interviews conducted via interpreter, Sarah Adolphe (Pan-American Health Organization). Additionally, in mother participants, quantitative methods utilized validated surveys including the PCL-C for PTSD screening, PHQ-9 for depression screening, maternal postpartum quality of life (MAPP-QOL) survey, and demographic information all captured using REDCap (Research Electronic Data Capture) database, hosted at Icahn School of Medicine at Mount Sinai, on a tablet provided to mothers [6]. All surveys were provided in Kreyol only after the option to complete the surveys verbally with the interpreter was offered.

**Table 1:** Mother interview questions.

All mothers		Separated mothers only

*Can you describe your home environment?*	*How much does maternal-child separation occur in your community?*	*Why did you separate from your child?*
*Can you describe your family’s circumstances at the time of your child’s birth?*	*Do you have any family or friends who have separated from their children? If yes: What do you think the decision to separate from their child was like?*	*How do you feel about the decision to separate from your child?*
*Can you describe your community’s opinion regarding your family’s situation?*	*What are factors that you think contribute to mothers separating from their children? Prompts: health (mother and child), finances, family/social support, religion?*	*How did you reach that decision? Were there outside factors? If so, what were they?*
*Can you tell me about your relationship with your parents?*	*Do you use birth control or contraceptives? Why or why not?*	*Did anyone help you make the decision? If so, who?*
*Were you separated from your mother as a child?*	*Was/were your pregnancy/pregnancies intentional or wanted?*	*Did the child’s health contribute to abandonment?*
*Can you describe your current emotional state?*	*Do you feel as though there has been any pressure in making decisions about child’s future?*	*Did the family’s financial situation contribute to abandonment?*
		*Did social/family support contribute to abandonment?*

**Table 2:** Community leader interview questions.


*How much does child relinquishment occur in your community?*	*Can you describe their families’ circumstance at the time of the birth of their child?*	*Are you aware of whether or not the pregnancy was intentional/wanted?*
*What do you think the decision to separate from a child is like for the mother?*	*Can you describe the community’s opinion regarding the family’s situation?*	*Do you believe that there is access to family planning or contraceptives?*
*Do you feel you have the resources to impact this?*	*Can you describe the relationship with their family members (mother and father)?*	*Do you feel as though there was any pressure in making the decisions in regard to their child’s future?*
*What resources do you wish you had?*	*Do you know if they were separated from their mothers as a child?*	*What are factors that you think contribute to mothers relinquishing their children?*
*Please think of a member or members of your community who have separated from their children or child for the following questions:*	*Can you describe their home environment?*	


This study employed the “snowballing/chain referral” method for identifying participants which is particularly appropriate for studies that touch on sensitive and stigmatized topics such as maternal-child separation [7]. Each interview was concluded by asking the participant for recommendations of community leaders who had experience with or exposure to maternal-child separation, as well as mothers who had and had not separated from a child. Community leader sampling ceased when responses became saturated, i.e. the same individuals were being referred by each interviewee. We worked with local Haitian directors of HaitiChildren to aid our search and referral process.

### Qualitative Data Analysis Methods

Interview recordings were transcribed nightly. We used conventional content analysis to analyze the interviews. This method was chosen because there are no publications regarding motivations for maternal-child separation in this region and to avoid limiting our analysis to preconceived categories given the broad range of information provided and the semi-structured nature of the interviews. We separately read and re-read all interview transcripts. After immersing ourselves in the data, we separately identified common broad themes related to maternal-child separation and apparently unrelated themes which were strongly represented in the data. We then reviewed this coding, discussed, and reconciled any discrepancies.

### Quantitative Data Analysis Methods

SAS 9.4 (SAS Institute Inc., Cary, NC) was used to analyze the bivariate and univariate distribution of variables captured in the PCL-C, PHQ-9, MAPP-QOL and demographic data. Additionally, given the non-normal distribution, Wilcoxon rank sum was utilized to examine the difference in means between separated and non-separated mothers. First, the given surveys were totaled as detailed in the description of the PCL-C, PHQ-9, and MAPP-QOL. The sums of each survey were then observed and stratified by status of separation, in order to identify mean differences between the two groups of mothers. It should be noted that the majority of mothers were not separated mothers (separated n = 8 and non-separated n = 41) limiting the potential for further factor analysis given the limited number of cases and lack of adequate sample size.

The study was conducted with support from HaitiChildren and Icahn School of Medicine at Mount Sinai (ISMMS) with approval from the ISMMS Program for the Protection of Human Subjects and the Haitian Ministry of Public Health.

## Findings

70 interviews (8 separated mother, 44 non-separated mother, and 18 community leader) were completed with 79 individuals (8 separated mother, 48 non-separated mother, and 23 community leader). Completed quantitative information was collected from 49 mothers (8 separated, 41 non-separated). Characteristics of the study sample are summarized in Tables 3 and 4. Themes are outlined in Table 5.

**Table 3:** Individual, Interview, and Quantitative Information Types.

	Individuals	Interviews	Complete quantitative information

**Mothers**	56	52	49
Separated	8	8	8
Non-separated	48	44	41
**Community leaders**	23	18	
Female	11		
Male	12		
NGO affiliated*	13	11	
Government affiliated	6	5	
Religious organization*	6	4	
**Total**	79	70	49

* Some interviewees were affiliated with a religion based NGO and as such are counted twice in this table.

**Table 4:** Maternal Characteristics.

	N (%)	Mean

**Age**		39.98
**Number children**		3.22
**Number prior births**		5.18
**Marital status**		
Married	13 (23.3%)	
Live with partner	10 (17.8%)	
Single	22 (39.3%)	
Separated	5 (8.9%)	
Widowed	6 (10.7%)	
**Ever attended school**	47 (83.9%)	
**Child with disability**	9 (16.1%)	

**Table 5:** Summary of themes.


*Economic means is the most commonly mentioned factor contributing towards maternal-child separation*.	*Education surrounding and use of family planning measures is sparse*.
*Means to provide education for the child is the next most commonly mentioned factor contributing towards maternal-child separation after economic means*.	*While not cited as a factor in maternal-child separation, having a disabled child was repeatedly mentioned as a source of additional pressures, both social and financial*.
*Lack of support from, absence of, or rejection by the partner/spouse is a contributing component to maternal-child separation*.	*Women reported fear of judgement from their communities*.
*Lack of support system contributes to maternal-child separation*.	*Maternal mental health is poor overall, particularly in mothers separated from their children*.
*There is a pervasive negative stigma towards maternal-child separation*.	


### Quality of Life

#### Economic means is the most commonly mentioned factor contributing towards maternal-child separation

Respondents cited lack of economic security as a burden facing parents in the Port-Au-Prince metropolitan area. Community leaders, as well as case and control mothers, expressed the influence of lack of economic means in the decision to separate from a child in both interviews and surveys.

“Sometimes a mother doesn’t have the resources to take care of the kid.”

“For a mother to make the decision to abandon their kids is very difficult but is linked to that they are not able to provide the minimum for their children and it is always for economic reasons.”

“Some are very sad about it because they have the mother instincts, but some only have the survival instincts… They are so poor and they have to fight every day for survival.”

**Figure 1:**
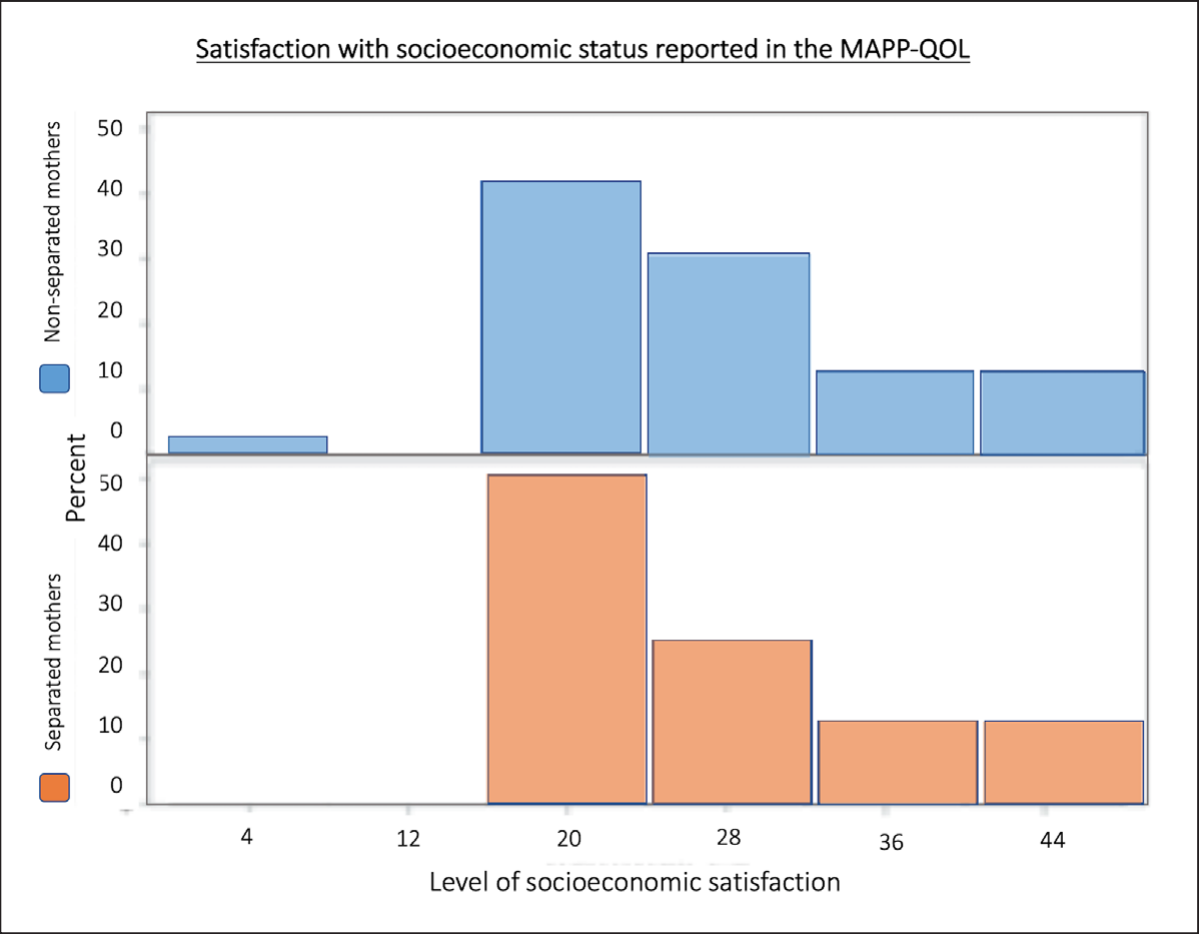
**MAPP-QOL Socioeconomic Satisfaction Distribution**. Socioeconomic status sum within the MAPP-QOL (Maternal Postpartum Quality of Life Survey) was not found to be significantly different between separated and non-separated mothers.

The MAPP-QOL socioeconomic subsection asks mothers to rate their level of satisfaction with living conditions, financial independence and capacity, ability to meet financial obligations, employment, and access to healthcare and transportation. While the above qualitative findings implicate economic means, socioeconomic status as measured by the MAPP-QOLwas not found to be significantly different between separated and non-separated mothers. This is seen in the graphic distribution (Figure 1) showing that non-separated mothers in fact reported worse economic status than separated mothers.

#### Means to provide education for the child is the next most commonly mentioned factor contributing towards maternal-child separation after economic means

Case and control mothers alike discussed the value of providing formal education for their children and the simultaneous economic strain. Approximately 90% of schools in Haiti are privately run, and as such, the average annual cost of school is 130 USD [8]. Community leaders expressed the impact of education on maternal-child separation, and separated mothers similarly provided tuition as an important factor in their decision to separate from their child.

“They were at school, then came summer vacation, and I saw that I wouldn’t have the resources to pay school for them… So I put them in the orphanage… When they finish school, the orphanage will give them back to me… When I put my children here, I felt like they were helping me. They are supporting me, because they teach them.”

“I didn’t have the resources to take care of my children to send them to school, so I decided to leave them [in the orphanage]. I didn’t have a chance to go to school, so I didn’t want my children to turn out like me, to not be able to go to school. So I thought it would be good for them to be [in the orphanage].”

“Compulsory free primary education would help solve a lot of problems, with the mandate that it be inclusive and that children with disabilities had equal access to it.”

#### Lack of support from, absence of, or rejection by the partner/spouse is a contributing component to maternal-child separation

**Figure 2:**
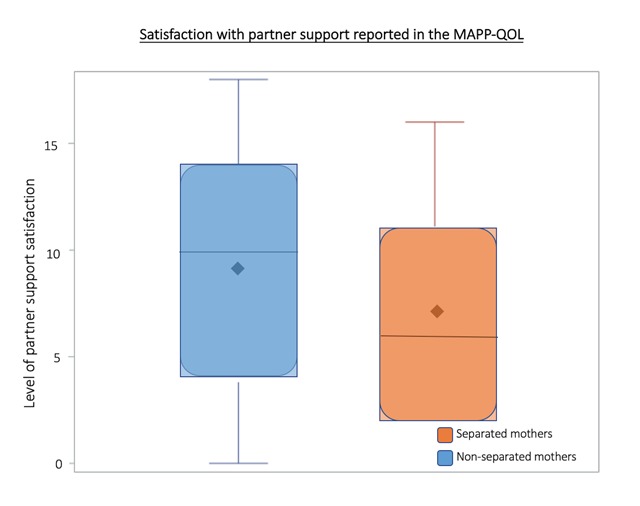
**MAPP-QOL Partner Support Satisfaction Distribution**. The shaded box represents the interquartile range (IQR), the range from the 25th percentile (Q1) to the 75th percentile (Q4). Median and mean are indicated by solid line and diamond, respectively. Horizontal lines indicate minimum and maximum values when they fall outside of the IQR. A greater score indicates a greater feeling of satisfaction. The partner scores were found to have a large difference between separated and non-separated mothers in terms of the magnitude of effect reported when utilizing a wilcoxon rank sum test, with a p-value of <0.01. (Figure 1). Thus, lack of partner support is associated with maternal-child separation.

The MAPP-QOL partner/spouse subsection asks mothers to describe their satisfaction with their partner/spousal support and home environment. Greater scoring indicates a greater feeling of satisfaction. The partner scores were found to be significantly different between separated and non-separated mothers with a p-value of <0.01. (Figure 2). The burden of poor paternal support was echoed in interviews. Partner support or lack thereof impacts maternal quality of life and adds to the complexity surrounding the decision to separate from a child.

“The other cases that present very often are of fathers who do not take up their responsibility. The fathers say they are not willing to take care of the child. If the father leaves, the woman at a certain point may know she cannot take care of the child.”

“The biggest factor is abandonment of the father in the first place.”

“They lived in a very disadvantaged area and is located in the south side of Port-Au-Prince known as a no man’s land. In that area there are particular issues of insecurity, particularly money, shelter, and food. So in that case the women had no means necessary and did not even know who the father was.”

#### Lack of support system contributes to maternal-child separation

When prompted to consider social and family support in the role of maternal-child separation, mothers and community leaders overwhelmingly agree that these forms of support are crucial in a mother’s decision. The interview question was intentionally open-ended such that participants could address any form of support. Several participants suggested lack of support in the form of social services is contributory to maternal-child separation.

“I didn’t have anyone to help me, to support me, so I had to put my kid in the orphanage.”

“Most of the time, the person doesn’t have any support from her family or from the society, so that’s usually why she decides to abandon her child.”

“There is no society to help a mother in need and if she had social support she wouldn’t have come to this decision [to abandon].”

“When my husband was still alive… he could look after the children. Since he is not around anymore and I need to go out every day to work, I cannot look after them. If they keep going outside to the neighbors, they keep playing, everyday people come to tell her things they have done. Sometimes even if they didn’t do something, [the neighbors] accuse them… So I decided not to keep them anymore, to put them in an orphanage, because I cannot spend my days looking after them.”

#### There is a pervasive negative stigma towards maternal-child separation

Despite the relative frequency of maternal-child separation in Port-Au-Prince, maternal-child separation retains a negative association as seen in other parts of the world. A negative stigma towards the concept of maternal-child separation manifests in variable ways. Few mothers and community leaders openly express disdain for mothers who separate from a child. Many distanced themselves from maternal-child separation by understating their knowledge of or relationship to separated families. Several participants described varying definitions of the word “abandone” allowing for non-traditional living arrangements to be considered more socially acceptable while elucidating how a wide range of living situations may or may not be considered separation based on the intention of the mother.

“Once you give birth to a kid, you should find any way to take care of him, find any job to take care of him. Lack of [social and family] support shouldn’t be a reason why [a mother abandons], because you are responsible for that kid.”

“I haven’t met any mothers who have abandoned their kids… [My neighbor] called me downstairs and told me she was having a baby and needed help… I decided if [the baby is] alive, [my neighbor] can’t just kill it… I tried to find a place to take the baby, but the orphanage said the baby is too young so I kept him for a while.”

“In Haiti, culturally children are a benediction, a blessing, and in a lot of disadvantaged communities, children are a blessing and the child is hope to bring them out of where they are… In good Kreyol, it means picking that family up from the ground. According to the community, their perception of a person abandoning the child is very negative.”

#### Education surrounding and use of family planning measures is sparse

In Haiti, a frequently discussed and accessible form of contraception is a contraceptive injection (medroxyprogesterone, brand name Confiance), referred to as “planning.” These findings suggest great concern regarding a wide range of potential and perceived adverse events from the contraceptive injection. In the event that mothers are unable to use the injection, many resort to having unprotected sex due to the perception that there are no other available methods or that intermittent use may be sufficient to prevent pregnancy. In addition to the sparse use of contraception, many mothers report unplanned pregnancies or state that the number of “planned” pregnancies exceeds the number of children they had hoped to have. The latter is more frequently the case with married mothers who describe the marriage itself as a plan to have children.

“I used to use planning but I am allergic to it… I used an injection called Confiance…. I didn’t plan to have children, so they were all accidents. I was pregnant 8 times. I had 2 abortions and lost 1 and gave birth to 5.”

“I know where to get it. It’s accessible and I have the resources, but I am not on a contraceptive. Because I don’t want to gain weight, because the injections that they give [at the clinic] make me gain weight.”

“I think that women who need to have family planning or contraception are women who are married or live with a partner. Since I don’t live with my partner, I don’t use any contraceptives. Once in a while, I think about it. When I have time, we have sex, so I don’t need any contraception… Since I already have a kid, I love him, so I will stay with him… but the next baby I will have, I will make sure to get married and follow everything the bible says before having another baby.”

“[Pregnancy is] only not accident when you married. When you married it’s welcoming but when you not married it’s a whole different thing.”

#### While not cited as a factor in maternal-child separation, having a disabled child was repeatedly mentioned as a source of additional pressures, both social and financial

Child disability was not found to be a factor in maternal-child separation. However, it was described as financially burdensome, a cause for community judgement, and a contributing factor in paternal absence. These associations, while not found to be a contributing influence in a mother’s decision to separate from a child, demonstrate the complexity of being a parent with a disabled child in Port-Au-Prince.

“We are in a society where there is no real good access to public healthcare, there is no insurance really, and the financial costs with a child with a disability or chronic medical condition will drown a family financially indefinitely… ‘If I keep this child, I have to keep paying for speech, or wheelchairs, or rehab, or whatever that is and I can no longer pay for school for my other children,’ and sometimes it’s a matter of trying to make the best out of a terrible situation.”

“In case of the child being born handicapped, the mother receives pressure from the father saying, “No this is not my child. This is your child.” Because the child is handicapped. Eventually humiliation from other people in the community will have that woman abandon that child even if she wanted to keep that child.”

#### Women reported fear of judgement from their communities

Women participating in the survey described fear that community members would perceive their struggle, and that this fear affects their decisions. No participants openly expressed judgement towards struggling community members, though several mothers distanced themselves from neighbors in efforts to conceal the magnitude of their own struggles. Many discussed the experience of or the fear of being judged.

“I wake up every morning and go to my small business, because I need the money to handle my stuff, to take care of myself, and because I can’t go to church dressed any-how. I need to be dressed to impress so people don’t know how I am living.”

“When life is easy for us, we are happy. When it’s not, we deal with it and we pray that it will get better… I used to go to church regularly, but since I started to have a lot of problems I stopped, because I cannot go to church dressed neglectfully.”

“Everybody handles their own stuff. Nobody goes to the neighbors, and everybody stays at their place to handle their own things.”

### Psychological Measures

#### Maternal mental health is poor overall, particularly in mothers separated from their children

### PHQ-9

**Figure 3:**
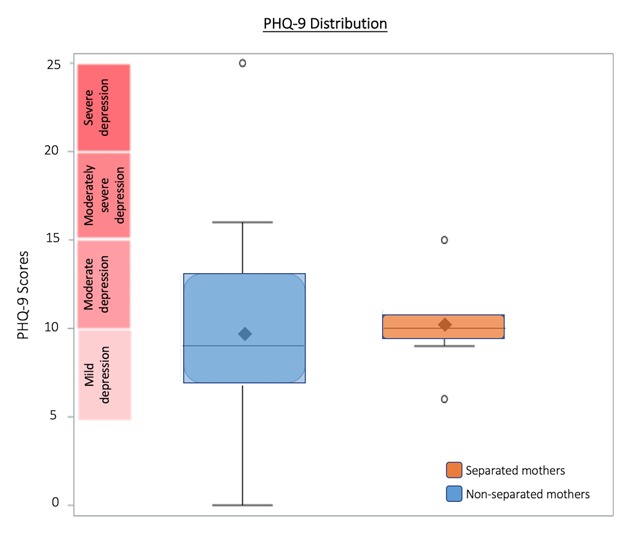
**PHQ-9 Distribution**. The shaded box represents the interquartile range (IQR), the range from the 25th percentile (Q1) to the 75th percentile (Q4). Median and mean are indicated by solid line and diamond, respectively. Horizontal lines indicate minimum and maximum values when they fall outside of the IQR. Open circles indicate outliers as defined by less than Q1–(1.5*IQR) or greater than Q3+(1.5*IQR). PHQ-9 scores indicate mild-moderate depression in 76% of mothers. PHQ-9 scores were not found to be statistically different between those who were separated and those were not.

The PHQ-9 depression severity scoring validity has been assessed against an independent structured mental health professional (MHP) interview. PHQ-9 score ≥10 had a sensitivity of 88% and a specificity of 88% for major depression. Of all 49 mothers who completed the PHQ-9 survey, approximately 76% (37 mothers) received scores indicating mild to moderate depression. However, PHQ-9 scores were not found to be statistically different between separated and non-separated mothers (Figure 3).

### PCL-C

**Figure 4:**
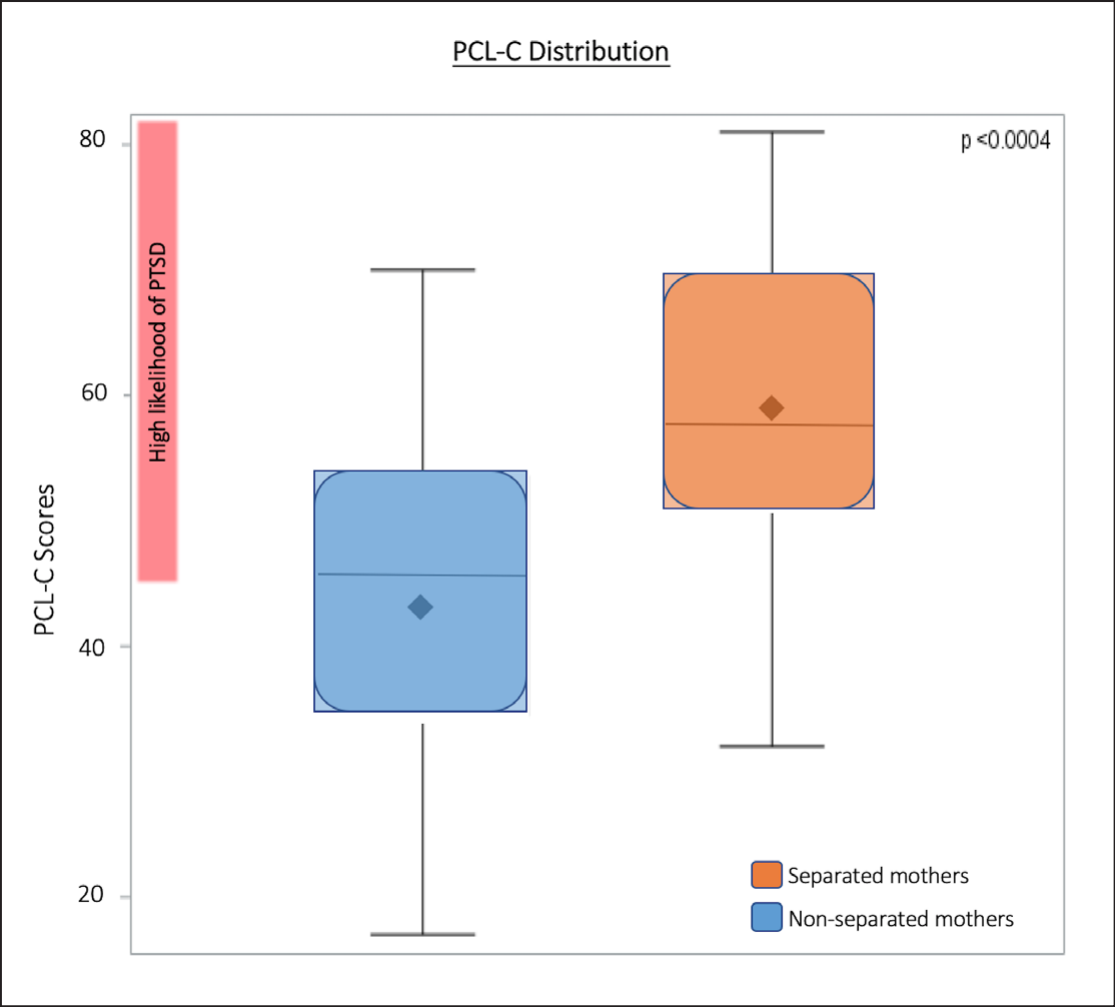
**PCL-C Distribution**. The shaded box represents the interquartile range (IQR), the range from the 25th percentile (Q1) to the 75th percentile (Q4). Median and mean are indicated by solid line and diamond, respectively. Horizontal lines indicate minimum and maximum values when they fall outside of the IQR. 58.3% of mothers overall scored >44 on the PCL-C, indicating high potential of PTSD. PCL-C scores are significantly higher in separated mothers, with over 80% of separated mothers scoring >44.

Potential scores range from 17 to 85. Scores of above 44 indicate a high likelihood of PTSD. Of the total 49 mothers who completed the PCL-C survey, 58.3% scored over 44. PCL-C scores are significantly different between separated and non-separated mothers with over 80% of separated mothers scoring over 44 indicating likely PTSD (p < 0.0004) (Figure 4).

### MAPP-QOL: Psychological subsection

**Figure 5:**
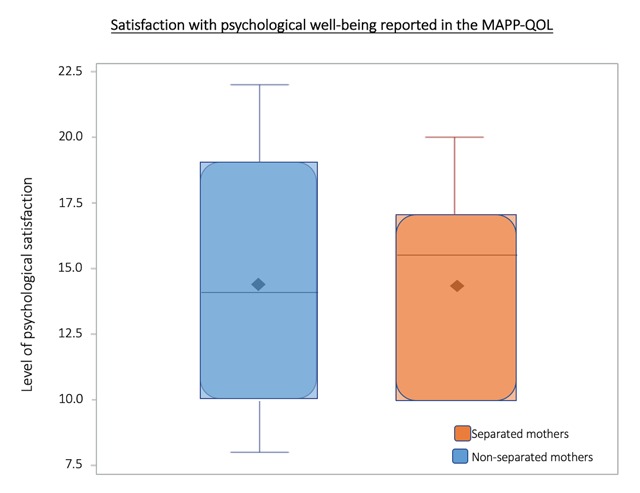
**MAPP-QOL Psychological Satisfaction Distribution**. The shaded box represents the interquartile range (IQR), the range from the 25th percentile (Q1) to the 75th percentile (Q4). Median and mean are indicated by solid line and diamond, respectively. Horizontal lines indicate minimum and maximum values when they fall outside of the IQR. Open circles indicate outliers as defined by less than Q1–(1.5*IQR) or greater than Q3+(1.5*IQR). A greater score indicates a greater feeling of satisfaction. The psychological scores were significantly different between separated and non-separated mothers, however, this difference was relatively small.

The MAPP-QOL psychological subsection asks mothers to describe their satisfaction with the amount of control they have over their lives, their peace of mind, their general happiness, their daily lives, and other such questions addressing overall mood. A greater score indicates a greater feeling of satisfaction, and a lower score indicates lack of satisfaction. The psychological scores were found to be significantly different between separated and non-separated mothers, however, this difference was relatively small (Figure 5).

## Discussion

This study found economic burden to be the most commonly cited factor contributing to maternal-child separation per qualitative methods, though quantitative findings suggested otherwise. Economic burden is likely a necessary, but not sufficient, factor in predicting maternal-child separation in Port-Au-Prince, Haiti. These findings suggest separation is associated with poor economic means when compounded with other factors including limited access to education, insufficient support (from child’s father and social network as well as absence of structural support), and poor maternal mental health. Additional findings of this study include limited use of family planning, stigma towards maternal-child separation, and the economic and social burden of having a disabled child.

It should be noted that many participants in this study emphatically expressed that *Haitian culture upholds children as a blessing to all families* and that circumstances surrounding maternal-child separation are complex and emotional. Mothers and community leaders alike expressed that the decision to separate is not made lightly and is a consequence of necessity.

Our findings are consistent with previous research that highlights the multifactorial situation surrounding maternal-child separation in other low-income countries. A UNICEF study in Vietnam found that poverty was the self-reported primary reason for mothers initiating separation but was insufficient in predicting separation. Rather poverty was associated with separation only when compounded with other factors including lack of social support, lack of access to social assistance, paternal absence, or unwanted pregnancy. In such situations, “the idea or suggestions from others to place a child either in an institution or to relinquish him or her for adoption were perceived as a window of opportunity [9].”

Limitations of this study include the small number of separated mothers. The relatively small number of separated mothers included in the study caused our analysis to weigh each of their responses more heavily, possibly biasing the results away from the null. When considering difficulties with recruiting separated mothers, it is worth noting that the small proportion of participants reporting separation contrasts with the large volume of children in Haiti’s orphanages that have at least one living parent. This dissonance raises questions about stigma and the reluctance to disclose separation. In order to greater power our findings, further studies with larger sample sizes are necessary.

Despite limitations, this study provides evidence to support possible interventions to support families in the Port-Au-Prince area. The prenatal period is likely optimal for screening and connecting high-risk mothers to resources to reduce separation in the immediate sense. An additional potential point of intervention identified is education surrounding family planning to reduce unplanned pregnancies. A randomized controlled trial in Nepal found that a 20-minute health education session for mothers directly following childbirth lead to increased likelihood of contraceptive use six months postpartum [10]. A standardized educational intervention with emphasis on family planning should be considered for mothers in Port-Au-Prince in the immediate postnatal period, whether in home or hospital settings, to mitigate risk of future unplanned pregnancies.

Another point of intervention identified is improvement of maternal mental health, particularly PTSD and depression. Psychological and psychiatric intervention for mothers and families could improve maternal mental health at the individual level. In addition, community-level intervention should be considered to address social determinants of mental health. A World Health Organization meta-analysis identified social determinants of perinatal mental health including poor or overcrowded living conditions, community violence, and social instability [11]. In low-income neighborhoods within high-income countries, neighborhood regeneration addressing some of the aforementioned social determinants of mental health has been associated with community-wide improvement in mental health. In one study, domains of regeneration included crime, education, health, housing and physical environment, vocational training and business support, and community [12]. The Emerging Mental Health Systems in LMICs (low- to middle-income countries) program (Emerald) aims to improve mental health outcomes in six LMICs by “generating evidence and capacity to enhance health system performance [13].” Emerging findings of the Emerald program may be helpful in developing system processes to improve mental health conditions for mothers in Port-Au-Prince.

Our findings point towards better access to schooling, for both children and mothers, as critical in prevention of maternal-child separation. Lack of access to education was cited as a major reason for maternal-child separation by majority of participants. Free or low-cost schools could address children’s access to education [14]. These schools could additionally address concerns raised regarding lack of education about birth control, lack of support, stigma, and presumed lack of community understanding about a mother’s decision to separate. Schools provide a place where parents could gather for any range of services including education, reproductive health, and access to contraception. Schools can be places where communities gather, learn about one another, and support one another. Finally, schools can be sources of employment for communities, thereby potentially indirectly reducing the financial burden on some otherwise separation-inclined mothers.

Lastly, efforts should be made to streamline and reallocate international aid that supports the orphanage system to resources that support family stability and reunification such as free education, school meals, and daycare facilities, which would ease the financial burden on parents while allowing them time to pursue employment. Efforts of many NGOs and the Haitian government have created a complex landscape of resources in Port-Au-Prince that must be adequately navigated to support mothers and children and mitigate risk of maternal-child separation.

Future directions involve researching the generalizability of these findings to the population of Port-Au-Prince. To the extent that factors identified in this study are generalizable, the challenge will be finding ways of remedying vast and endemic issues such as poverty and access to schooling and studying their impact on maternal-child separation. Such studies would likely need to occur on a small scale, at the level of neighborhoods. This will likely introduce practical and ethical challenges. Regardless of future findings on the topic of maternal-child separation, remedying social ills like poverty or lack of education would constitute accomplishments in themselves with dividends extending incalculably far beyond maternal-child separation.
